# Comparative HPLC–DAD–ESI-QTOF/MS/MS Analysis of Bioactive Phenolic Compounds Content in the Methanolic Extracts from Flowering Herbs of *Monarda* Species and Their Free Radical Scavenging and Antimicrobial Activities

**DOI:** 10.3390/pharmaceutics15030964

**Published:** 2023-03-16

**Authors:** Małgorzata Kozyra, Anna Biernasiuk, Magdalena Wiktor, Wirginia Kukula-Koch, Anna Malm

**Affiliations:** 1 Department of Pharmacognosy with the Medicinal Plants Garden, Medical University of Lublin, 1 Chodźki Str., 20-093 Lublin, Polandvirginia.kukula@gmail.com (W.K.-K.); 2 Department of Pharmaceutical Microbiology, Medical University of Lublin, 1 Chodźki Str., 20-093 Lublin, Poland; anna.biernasiuk@umlub.pl (A.B.); anna.malm@umlub.pl (A.M.)

**Keywords:** *Monarda* spp., HPLC–DAD–ESI-QTOF/MS/MS, flavonoids, phenolic acids, antioxidant, antimicrobial

## Abstract

Comparative analysis of flavonoids and phenolic acids composition, in plants of six species of *Monarda* from family Lamiaceae was carried out. The 70% (*v/v*) methanolic extracts of flowering herbs of *Monarda citriodora* Cerv. ex Lag., *Monarda bradburiana* L.C. Beck, *Monarda didyma* L., *Monarda media* Willd., *Monarda fistulosa* L. and *Monarda punctata* L. were analyzed for their polyphenol composition as well as antioxidant capacity and antimicrobial effect. Liquid chromatography-electrospray ionization-tandem mass spectrometry (HPLC–DAD–ESI-QTOF/MS/MS) was used to identify phenolic compounds. The in vitro antioxidant activity was assessed using a DPPH radical scavenging assay, while antimicrobial activity was measured by the broth microdilution method allowing for MIC (minimal inhibitory concentration) determination. The total polyphenol content (TPC) was assayed by the Folin–Ciocalteu method. The results showed the presence of eighteen different components including phenolic acids and flavonoids together with their derivatives. The presence of six constituents (gallic acid, hydroxybenzoic acid glucoside, ferulic acid, *p*-coumaric acid, luteolin-7-glucoside and apigenin-7-glucoside) was found to be dependent on the species. To differentiate the samples, the antioxidant activity of 70% (*v/v*) methanolic extracts was studied and expressed as a percent of DPPH radical inhibition and in EC_50_ values (mg/mL). The latter values were as follows: *M. media* (EC_50_ = 0.090 mg/mL), *M. didyma* (EC_50_ = 0.114 mg/mL), *M. citriodora* (EC_50_ = 0.139 mg/mL), *M. bradburiana* (EC_50_ = 0.141 mg/mL), *M. punctata* (EC_50_ = 0.150 mg/mL) and *M. fistulosa* (EC_50_ = 0.164 mg/mL). Moreover, all extracts indicated bactericidal activity against reference Gram-positive (MIC = 0.07–1.25 mg/mL) and Gram-negative bacteria (MIC = 0.63–10 mg/mL) as well as fungicidal effect towards yeasts (MIC = 1.25–10 mg/mL). *Staphylococcus epidermidis* and *Micrococcus luteus* were the most sensitive to them. All extracts showed promising antioxidant properties and noteworthy activity against the reference Gram-positive bacteria. Antimicrobial effect of the extracts against the reference Gram-negative bacteria as well as fungi (yeasts) from *Candida* spp. was slight. All extracts showed bactericidal and fungicidal effect. The obtained results indicated that the investigated extracts from *Monarda* spp. could be potential sources of natural antioxidants and antimicrobial agents, especially with activity towards Gram-positive bacteria. The differences in the composition and properties of the studied samples may influence the pharmacological effects of the studied species.

## 1. Introduction

*Monarda* taxa are widely known in modern and traditional medicine. The *Monarda* genus (common name bee balm) belongs to the Lamiaceae botanical family and includes about 16 species of herbaceous plants native to North America. Nowadays, representatives of the studied genus grow also in Europe. They are particularly used in the treatment of digestive disorders and bronchial ailments. The flowering herbs also contain anthelmintic, expectorant, carminative, rubefacient, diuretic, pyrogenic activity, stimulant and strong antiseptic ingredients suitable for the treatment of skin infections and minor wounds [1,2,3,4,5]. Essential oils (EOs) are the main group of biologically active substances which were described in *Monarda* spp. [3,6,7]. In turn, thymol is the major monoterpene phenol occurring in EOs isolated from plants belonging to the Lamiaceae family, including the *Monarda* genus. Moreover, the antifungal and antioxidant activity of EOs extracted from some *Monarda* species were also described [1,8].

Polyphenols constitute the other group of biologically active compounds of *Monarda* spp. [4,5,9,10,11,12,13,14]. It is well known that polyphenols are a large and diverse group of secondary metabolites playing many essential roles in plant physiology. These compounds are characterized as bioactive ingredients, including antioxidant, antiallergic, anti-inflammatory, anticancer, antihypertensive and antimicrobial agents [6,7,15,16,17,18,19,20]. *M. didyma* leaves and flowers were found to include polyacylated anthocyanins containing coumaric and malonic acids and the flavonols rutin, hyperoside, quercitrin, luteolin and quercetin in a high-performance liquid chromatography (HPLC) study. The authors noted that the quantity of flavonoids was higher in the flowers than in the leaves [11,12].

From the petals of *M. fistulosa* anthocyanins, flavonoids and hydroxycinnamic acids, such as *p*-coumaric acid glucoside, 7,4’-dihydroxyflavone 8-*C*-glucoside, flavone-7-*O*-glucoside, acylated pelargonidin glycosides, diacylated pelargonidin-3,5-diglucoside, apigenin-7-*O*-glucoside and 5-hydroxyflavone, were isolated using the following hyphenated techniques: HPLC, column, paper, and thin-layer chromatography techniques by Davies and Mazza [13] and Marshall and Scora [14]. Flavones and phenolic acids like apigenin-7-*O*-rutinoside, rosmarinic acid, linarin, apigenin, acacetin were also present in the 70% hydro-ethanolic extract from the fresh and senescent flowers.

Although some data presenting the bioactivity and the chemical composition of *Monarda* species have already been published [1,2,3,4,5,6,7,11,12,13,14], there is a need for further investigation of these plant species, especially for the presence of less-studied phenolic compounds, responsible for antioxidant and antimicrobial activity. The aim of the present study was to analyze the profile of phenolic compounds in the 70% (*v/v*) methanolic extracts of flowering herbs of *M. bradburiana*, *M. citriodora*, *M. didyma*, *M. fistulosa*, *M. media* and *M. punctata* by liquid chromatography-electrospray ionization-tandem mass spectrometry (HPLC–DAD–ESI-QTOF-/MS/MS) together with the determination of the total phenol content (TPC). These plants were cultivated in the Medicinal Plant Garden, Department of Pharmacognosy, Medical University in Lublin, Poland. Moreover, antioxidant and antimicrobial activity in vitro of the *Monarda* spp. methanolic extracts was also assayed.

## 2. Materials and Methods

### 2.1. Plant Material

According to the seed exchange policy, the seeds of the following *Monarda* species, namely *Monarda citriodora* Cerv. ex Lag., *Monarda bradburiana* L.C. Beck, *Monarda didyma* L., *Monarda media* Willd., *Monarda fistulosa* L. and *Monarda punctata* L. were obtained from differently located botanical gardens (The Botanical Garden of Medicinal Plants of the Medical University of Wrocław, Poland; Karl-Franzens-Universität Botanical Garden, Graz, Austria; Hortus Botanicus Nationalis, Salaspils, Latvia; Botanical Garden of the University of Ferrara, Italy; Botanic Garden of Maria Curie-Skłodowska University, Lublin, Poland; Botanical Garden in Lodz, Poland). They were planted in different areas of the Medicinal Plants Garden that is a unit of the Department of Pharmacognosy, Medical University of Lublin, Poland, and grown until flowering. The flowering herbs were collected in July 2018. Voucher specimens numbered *Monarda* 01-07/2018, respectively, have been stored in the herbarium of the same unit at the Medical University of Lublin. Before the analyses, plant material was first authenticated by Prof. Kazimierz Głowniak (Medical University of Lublin), who is a specialist in plant taxonomy, using different available botanical keys, e.g., the WHO Plant list (World Flora Online), Key Gardens information (nativeplanttrust.org) and virtual plant atlases.

Flowering herbs of *M. bradburiana, M. citriodora, M didyma, M. fistulosa, M. media* and *M. punctata* were dried at 45 °C and then powdered and sieved (0.315 and 0.074 mm sieves). The procedure of preparation met the requirements of the Polish Pharmacopoeia VI.

### 2.2. Chemical Reagents

The standards of flavonoids, phenolic acids at purity exceeding 95% and DPPH were obtained from Sigma-Aldrich (St. Louis, MO, USA). All reagents used for the Accelerated Solvent Extraction (ASE, Dionex, Sunnyvale, CA, USA), radical scavenging assays and TPC were obtained from Avantor Performance Materials (Gliwice, Poland). The reagents for the RP-HPLC analysis (acetic acids, methanol) and for the HPLC–DAD–ESI/MS/MS analysis (acetonitrile, water and formic acid) were purchased from J.T. Baker (Deventer, Holland).

### 2.3. Microbiological Material

The examined extracts from *Monarda* spp. were screened for their antibacterial and antifungal properties in vitro using methods suggested by the European Committee on Antimicrobial Susceptibility Testing (EUCAST) [21] and Clinical and Laboratory Standards Institute (CLSI) [22] guidelines against a panel of 20 reference microorganisms, including Gram-positive bacteria (*Staphylococcus aureus* ATCC 6538, *Staphylococcus aureus* ATCC 25923, *Staphylococcus aureus* ATCC 43300, *Staphylococcus aureus* ATCC 29213, *Staphylococcus epidermidis* ATCC 12228, *Enterococcus faecalis* ATCC 29212, *Micrococcus luteus* ATCC 10240, *Bacillus subtilis* ATCC 6633 and *Bacillus cereus* ATCC 10876), Gram-negative bacteria (*Escherichia coli* ATCC 25922, *Bordetella bronchiseptica* ATCC 4617, *Klebsiella pneumoniae* ATCC 13883, *Proteus mirabilis* ATCC 12453, *Pseudomonas aeruginosa* ATCC 9027 and *Salmonella* Typhimurium ATCC 14028) and fungi belonging to yeasts (*Candida albicans* ATCC 10231, *Candida albicans* ATCC 2091, *Candida parapsilosis* ATCC 22019, *Candida krusei* ATCC 14243 and *Candida glabrata* ATCC 90030). The microorganisms belonging to ATCC came from American Type Culture Collection, which has been routinely used to study the antimicrobial potential. During the experiment all microbial cultures were subcultured on nutrient or Sabouraud agar for 18–24 h at 35 °C or for 24–48 h at the temperature of 30 °C for bacteria and fungi, respectively. Microbial suspensions were prepared in 0.85% NaCl (optical density of 0.5 McFarland standard), containing 1.5 × 10^8^ CFU/mL (Colony Forming Units/mL) for bacteria or 5 × 10^6^ CFU/mL for fungi.

#### 2.3.1. Preparation of Plant Extracts

Dried and powdered plant material, namely flowering herbs (1.0 g and 7.0 g) of the selected *Monarda* species, were subjected to extraction using the Accelerated Solvent Extraction in ASE 100 apparatus (Dionex, Sunnyvale, CA, USA). The conditions applied in the extraction process were as follows: extraction solvent: 70% methanol; number of cycles: 3; temperature: 85 °C; flush volume: 60%; purge time: 150 s and 10 min—duration of cycle method was optimized and described by Kozyra and Skalicka-Woźniak [23]. The obtained 70% methanol water extracts from (1.0 g) of *Monarda* spp. were later concentrated to dryness in a rotary evaporator at 50 °C under reduced pressure, and later re-dissolved in 70% methanol and filtered through a syringe filter (25 mm, 0.45 µm, PTFE Cronus Syringe Filter) into 10 mL volume calibrated vials. The obtained solutions were later analyzed for their composition by HPLC–DAD–ESI-QTOF-/MS/MS and later evaluated for antioxidant activity. The extracts obtained from 7.0 g of flowering herbs of *Monarda* spp. were evaporated in a rotary evaporator at 50 °C under reduced pressure, then dissolved in 1 mL of dimethyl sulfoxide and directed to in vitro antibacterial and antifungal activity screening using the broth microdilution method.

#### 2.3.2. HPLC–DAD–ESI-QTOF-MS/MS Analysis

The tested samples were analyzed qualitatively by an HPLC–DAD–ESI/MS/MS system composed of a 6530B Accurate-mass-QTOF/MS/MS mass detector (Agilent Technologies Inc., Santa Clara, CA, USA) with an ESI-Jet Stream ion source. The fingerprinting was performed in the negative ionization mode in the mass range from 100 to 1000 m/z. The platform used in the study was composed of an HPLC chromatograph equipped in a DAD detector, a binary gradient pump, an autosampler and a column oven. The Phenomenex (Torrance, CA, USA) Gemini^®^ C18 110 Å chromatographic column (dp = 3 μm, 100 × 2 mm I.D.) was used. The applied mobile phase used a gradient of two solvents, namely 1% acetonitrile in water with 10 mM ammonium formate and formic acid 0.1% (solvent A) and 95% aqueous acetonitrile with 10 mM ammonium formate (0.1%) and formic acid 0.1% (solvent B) in the following program: from 0–5 min 5–15% B; from 5–25 min 15–25% B; from 25–55 min 20–45% B; and from 55–70 min 45–95% B. Post time was set at 12 min, the flow rate at 0.2 mL/min and the sample injection volume was 10 µL. The total time of analysis was 70 min, the UV detection wavelengths applied were 254, 280 and 320 nm. The mass spectrometer was operated at the following conditions: negative ionization mode; gas temperature: 300 °C; gas flow rate: 12 L/min; sheath gas temperature: 350 °C; sheath gas flow rate: 12 L/min; nebulizer pressure: 35 psig; V Cap: 4000 V; octopole RF Peak: 750 V; skimmer: 65 V; and fragmentor: 140 V. The auto MS/MS acquisition mode was applied, and the collision-induced dissociation energies were 10 and 40 eV, the MS scan rate was set at 1 spectrum per second and 2 spectra per cycle. The qualitative fingerprinting was acquired in the auto MS/MS mode with two *m/z* excluded at 966.0007 and 112.9855 for the ions corresponding to the reference ions. The data were handled by the MassHunter (v. B.08.00) software (Agilent Technologies, Santa Clara, CA, USA). The identified phenolic components were compared with the scientific literature and the MS/MS fragmentation data present in the open databases, such as METLIN database [19,20,21,22].

#### 2.3.3. Determination of Total Polyphenolic Content (TPC)

The total polyphenolic content (TPC) assay was performed using Folin–Ciocalteu reagent based on the method described by Singleton and Rosi [24]. An amount of 200 μL of 70% (*v/v*) methanolic solution of dry extracts (1 mg/mL) was mixed with 400 μL (1 N) Folin–Ciocalteu reagent (Sigma-Aldrich, St. Louis, MO, USA) diluted with water (1:2) and after 5 min 3200 µL 75 g/L sodium carbonate (Na_2_CO_3_) was added. The mixture was well shaken at 20 °C for 20 min and the absorbance was measured at 760 nm. Moreover, gallic acid (Sigma-Aldrich, St. Louis, MO, USA) was used as the positive reference standard. The TPC content was in the end expressed as µg GAE (gallic acid equivalents) per mg of extract. The calculated equation that was obtained from the gallic acid graph was (R2 = 0.9926; y = 5.7095x). All tests were performed in triplicate.

#### 2.3.4. DPPH Radical Scavenging Activity

Antioxidant activity of the obtained extracts (1 mg/mL) was determined in a method of Brand-Williams et al. [25] by using the solution of 2,2-diphenyl-1-picrylhydrazyl radical (DPPH) in methanol (Sigma-Aldrich, St. Louis, MO, USA) (78 μg/mL). In the assay, the dilutions of 70% (*v/v*) methanolic of *Monarda* extracts were used. The tested samples were prepared in Eppendorf tubes to obtain the final concentrations of 1.5; 0.75; 0.375; 0.1875; 0.09375; 0.046875; 0.0234375 mg of per mL. In the assay, 20 µL of the extracts were mixed with 180 µL of the radical solution on a 96-well plate. The blank sample was composed of methanol instead of the extract solution and DPPH solution. After 30 min of incubation in the dark, the absorbance was measured at 515 nm (BioTek ELx808 plate reader, BioTek, Winooski, VT, USA). Computer program Gen5 version 2.01 was used for recording and handling the obtained data. All experiments were performed in triplicate. To compare the antiradical potential of the tested samples, the EC_50_ value was determined which indicates the inhibitory concentration of a sample at which 50% of DPPH radicals are being scavenged. This value was automatically calculated by the operational program of the platelet reader (4LP protocol) by using the following equation [26]:

Y = (A − D)/((1 + (X/C)^B)) + D

In the equation above, A was the theoretical response for concentration = 0, B was a measure of the curve’s slope at its inflection point, C was the x value at the inflection point, D—the theoretical answer for infinite concentration, X was the concentration parameter and Y—the calculated EC_50_ value.

To precisely assess the scavenging properties of the tested extracts, the EC_50_ value was also calculated (*n* = 3) for other compounds that are known radical scavengers, including Trolox, butylated hydroxytoluene (BHT), chlorogenic acid, gallic acid and caffeic acid. The obtained results are shown as average values ± standard deviation (SD). All the standard solutions and investigated samples of extracts, as well as the DPPH solutions, were prepared daily.

#### 2.3.5. Antimicrobial Assay

The determination of the antimicrobial properties of *Monarda* spp. extracts was performed in an in vitro method, using a diffusion assay in agar medium. For bacteria, the surface of Mueller-Hinton agar and for fungi, the RPMI 1640 with MOPS were inoculated with the microbial suspensions of fungal or bacterial species. Dried samples for testing were dissolved with DMSO to the concentration of 5 mg/mL. Next, 50 µL of them was transferred into the wells (d = 7 mm) on the agar media. The agar plates were pre-incubated for 2 h at room temperature and next they were incubated under the appropriate conditions corresponding to bacteria and fungi. When the incubation period was completed, the growth inhibition zones around the wells were measured. Negative controls were prepared with DMSO added instead of the tested samples. All the experiments were performed in triplicate and representative data are presented [27].

The MIC (Minimal Inhibitory Concentration) values of the tested extracts were determined by the microdilution broth method, by using their two-fold dilutions in the prepared Mueller-Hinton broth (for bacteria) and in the RPMI 1640 broth with MOPS (for fungi) that were prepared in 96-well polystyrene plates. In the assay, the final concentrations of extracts ranged from 20 to 0.15 mg/mL. The microbial suspensions for the assay were prepared in 0.85% NaCl with an optical density of 0.5 McFarland standard. Then, each bacterial or fungal suspension was added to each well containing broth and different concentrations of the tested extracts. Later, after incubation, the MIC value was assessed spectrophotometrically as the lowest sample’s concentration that showed a complete inhibition of bacterial or fungal growth. Appropriate DMSO content determination, growth and sterile controls were carried out as well. The medium with no addition of the tested extracts was also used as a control. In addition, luteolin (Sigma-Aldrich, St. Louis, MO, USA) was used as a reference natural antimicrobial compound. All experiments were performed in triplicate and representative data are presented [27,28].

The MBC (Minimal Bactericidal Concentration) or MFC (Minimal Fungicidal Concentration) are defined as the lowest concentrations of the tested samples that are required to kill a given bacterial or fungal species. MBC and MFC values were calculated by removing the cultures used for MIC determinations from each well and spotting them on an appropriate agar medium. The plates were incubated under appropriate conditions for bacteria and fungi. The lowest concentrations of the tested components with no visible growth of microorganisms observed were determined as bactericidal or fungicidal concentrations, respectively. The MBC/MIC or MFC/MIC ratios were later calculated in order to assess the bactericidal/fungicidal (MBC/MIC ≤ 4, MFC/MIC ≤ 4) or bacteriostatic/fungistatic (MBC/MIC > 4, MFC/MIC > 4) effects of the extracts. The procedure was repeated three times and the representative data are shown in the manuscript [27,28].

## 3. Results

### 3.1. Chemical Analysis of Phenolic Compounds in Methanolic Extracts from Monarda Species

In the present study, the composition of the 70% (*v/v*) methanolic extracts from flowering herbs of *M. citriodora*, *M. bradburiana*, *M. didyma*, *M. fistulosa*, *M. media* and *M. punctata* was determined. The HPLC–DAD–ESI-QTOF-/MS/MS was used to obtain their detailed chromatographic fingerprints and mass spectral information that could lead to a tentative identification of single components of the mixtures. Eighteen peaks of phenolic compounds were identified in these extracts by comparing their UV, MS and MS/MS spectra to those of the purchased analytical standards, the METLIN database and data from the literature [17,18,19,20,29,30].

**Figure 1:**
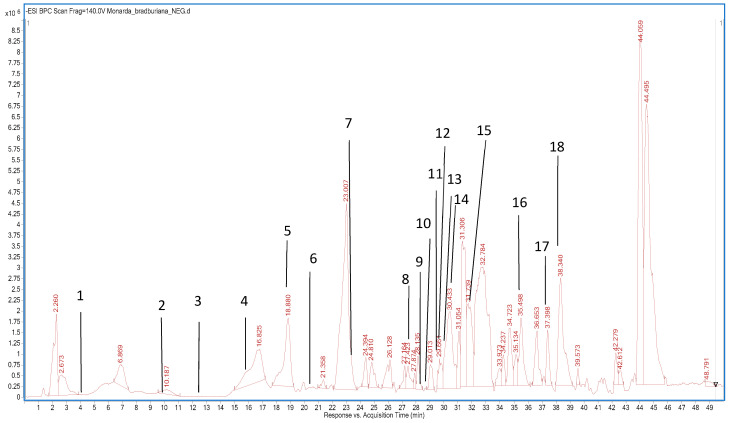
The HPLC–ESI-QTOF-MS/MS total ion current chromatogram of the 70% (*v/v*) methanolic extracts from flowering herbs of *M. bradburiana*: 1. gallic acid, 2. protocatechuic acid, 3. hydroxybenzoic acid glucoside, 4. *p*-OH-benzoic acid, 5. chlorogenic acid, 6. coumarylquinic acid, 7. *p*-coumaric acid, 8. kaempherol-3-rutinoside, 9. luteolin-7-glucoside, 10. naringin, 11. ferulic acid, 12. apigenin-7- rutinoside, 13. apigenin-7-glucoside, 14. luteolin-3-glucuronide 15. rosmarinic acid, 16. luteolin, 17. naringenin, 18. apigenin.

**Figure 2:**
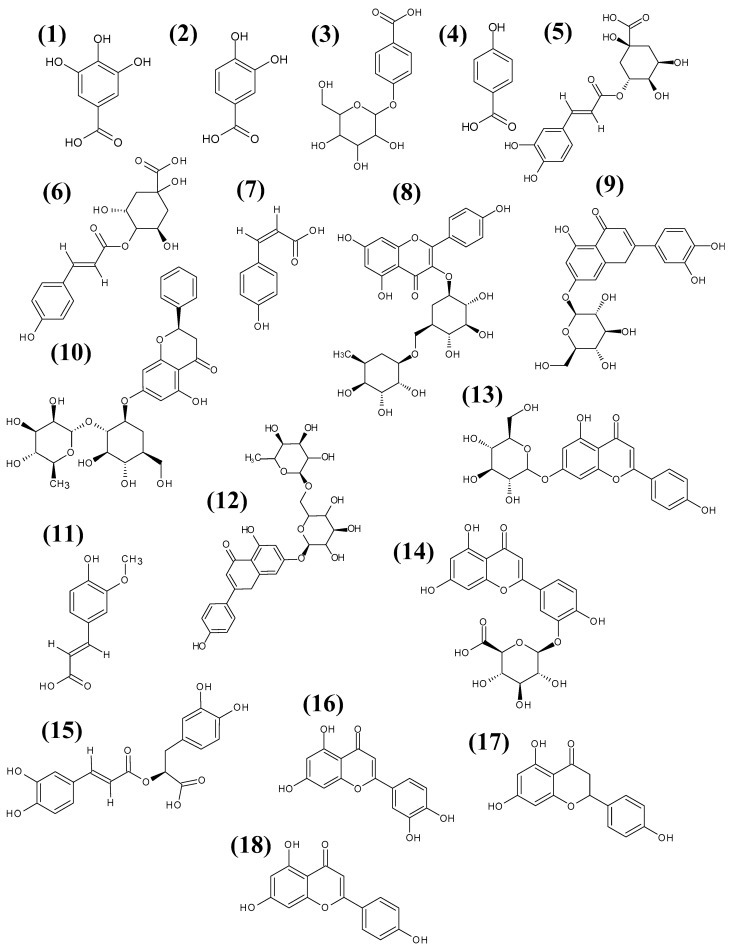
The chemical structures of the major components identified in the 70% methanolic extract from the flowering herbs of *M. bradburiana*: 1. Gallic acid, 2. Protocatechuic acid, 3. Hydroxybenzoic acid glucoside, 4. *P*-OH-benzoic acid, 5. Chlorogenic acid, 6. Coumarylquinic acid, 7. *P*-coumaric acid, 8. Kaempherol-3-rutinoside, 9. Luteolin-7-glucoside, 10. Naringin, 11. Ferulic acid, 12. Apigenin-7- rutinoside, 13. Apigenin-7-glucoside, 14. Luteolin-3-glucuronide 15. Rosmarinic acid, 16. Luteolin, 17. Naringenin, 18. Apigenin.

The results from Table 1, Figure 1 and their fragmentation spectra—in Tables S1 and S2 in the Supplementary file—showed the presence of 18 different components including both phenolic acids and their derivatives (chlorogenic acid, coumarinoquinic acid, *p*-OH-benzoic acid, protocatechuic acid, ferulic acid, gallic acid, rosmarinic acid, *p*-coumaric acid and hydroxybenzoic acid glucoside) as well as flavonoids and their derivatives (luteolin-3-glucuronide, kaempherol-3-rutinoside, luteolin-7-glucoside, apigenin-7-rutinoside, apigenin-7-glucoside, apigenin, luteolin, naringenin and naringin) in the studied extracts. The structures of the major components are presented in Figure 2 below. However, the number of ingredients in these extracts varied and were as follows: *M. fistulosa* (17), *M. bradburiana*, *M. citriodora* and *M. didyma* (16), *M. punctata* (15) and *M. media* (14). The profiles of six constituents were found to depend on the *Monarda* species: *M. bradburiana* (gallic acid, hydroxybenzoic acid glucoside, *p*-coumaric acid and ferulic acid), *M. citriodora* (gallic acid, hydroxybenzoic acid glucoside, *p*-coumaric acid and luteolin-7-glucoside), *M. didyma(* gallic acid, hydroxybenzoic acid glucoside, *p*-coumaric acid and apigenin-7-glucoside), *M. fistulosa* (gallic acid, hydroxybenzoic acid glucoside, *p*-coumaric acid, luteolin-7-glucoside and apigenin-7-glucoside), *M. media* (gallic acid and hydroxybenzoic acid glucoside) and *M. punctata* (ferulic acid, luteolin-7-glucoside and apigenin-7-glucoside).

### 3.2. The Total Polyphenol Content (TPC) in the Methanolic Extracts from Monarda Species and Their Antioxidant Potential

**Figure 3:**
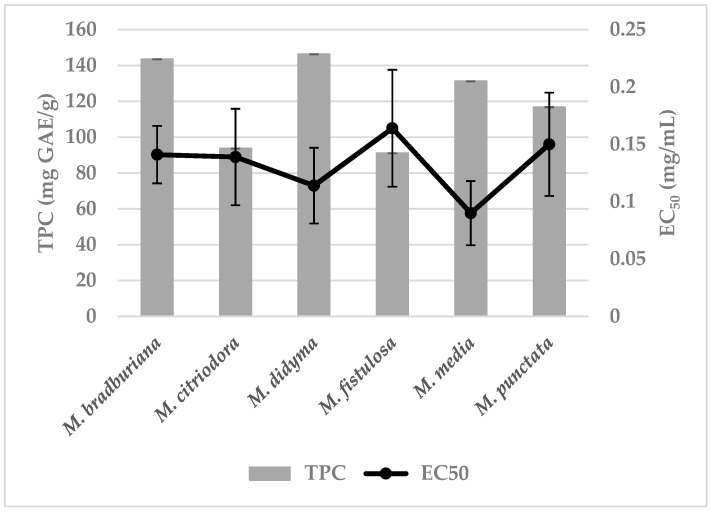
The total polyphenol content (TPC) calculated as gallic acid equivalents (GAE) in 70% (*v/v*) methanolic extracts of *Monarda* species, together with their antioxidant activity (EC_50_ value). Mean values ± SD were presented (*n* = 3).

As presented in Figure 3, the total polyphenol content (TPC) in 70% *v/v* MeOH extracts of *Monarda* species was determined by the Folin–Ciocalteu method and presented as gallic acid equivalents (GAE). The following values were obtained: 91.06 ± 0.047 mg GAE/g for *M. fistulosa*; 93.66 ± 0.052 mg GAE/g for *M. citriodora*; 116.76 ± 0.079 mg GAE/g for *M. punctata*; 131.23 ± 0.065 mg GAE/g for *M. media;* 143.50 ± 0.061 mg GAE/g for *M. bradburiana*; and 146.30 ± 0.036 mg GAE/g for *M. didyma*. The analysis of these results showed that the highest amount of polyphenols was found in the extracts of *M. bradburiana, M. didyma* and *M. media*.

A spectrophotometric study with the use of the DPPH reagent proved that the analyzed methanolic extracts from flowering herbs of *Monarda* spp. showed various antioxidant properties. Low values of the EC_50_ index, in the range of 0.090–0.164 mg/mL, constituted the high antioxidant potential of these species. The EC_50_ values for all obtained extracts were as follows: *M. media* (EC_50_ = 0.090 ± 0.028 mg/mL), *M. didyma* (EC_50_ = 0.114 ± 0.033 mg/mL), *M. citriodora* (EC_50_ = 0.139 ± 0.042 mg/mL), *M. bradburiana* (EC_50_ = 0.141 ± 0.025 mg/mL), *M. punctata* (EC_50_ = 0.150 ± 0.045 mg/mL) and *M. fistulosa* (EC_50_ = 0.164 ± 0.051 mg/mL) (Figure 3).

For comparison, several reference compounds that were proved to exhibit antiradical activity, such as gallic acid, caffeic acid, Trolox, butylated hydroxytoluene (BHT) and chlorogenic acid, were tested in alike conditions to show the strength of the tested extracts. The EC_50_ of the reference compounds were 0.023 ± 0.002 mg/mL, 0.051 ± 0.000 mg/mL, 0.089 ± 0.000 mg/mL, 0.112 ± 0.002 mg/mL, and 0.145 ± 0.004 mg/mL, respectively. The lowest value of the EC_50_ were found for the extracts from *M. media* and *M. didyma* which indicated their higher antiradical properties among the tested species—comparable to the Trolox solution.

### 3.3. The Antimicrobial Activity Assessment of Methanolic Extracts from Monarda Species

The antimicrobial activity of 70% (*v/v*) methanolic extracts from different species of *Monarda* was determined against the reference bacterial and fungal (yeasts) species. Assessment of these effect of extracts by the diffusion method indicated different growth inhibition zones of the reference microorganisms, as shown in Table 2 and Figure S1.

The diameters of the growth inhibition zones of Gram-positive bacteria ranged from 0 mm to 35 mm. Among them, the largest diameter zones of growth inhibition were found for *Bacillus subtilis* ATCC 6633 (11–35 mm). The diameters of growth inhibition zones of Gram-negative bacteria were found to range from 0 mm to 19 mm. Among these microorganisms, the most sensitive was *Bordetella bronchiseptica* ATCC 4617 (13–19 mm). Similar diameters of the growth inhibition were obtained for *Candida* species (0–17 mm). Among them, *Candida albicans* ATCC 10231 (12–13 mm) and *Candida krusei* ATCC 14243 (10–15 mm) were more susceptible to some extracts. For some microorganisms, the accurate measuring of the diameters of growth inhibition zones was difficult because single colonies were found within the zone or slight growth around the wells was observed.

Next, antimicrobial activity of the 70% (*v/v*) methanolic extracts of *Monarda* species was assessed on the basis of MIC (Minimum Inhibitory Concentration) and MBC (Minimum Bactericidal Concentration) or MFC (Minimum Fungicidal Concentration) as presented in Table 3, Table 4 and Table 5. As shown in Table 3, the values of MIC of the extracts against Gram-positive bacteria ranged from 0.07 mg/mL to 1.25 mg/mL depending on the *Monarda* and bacterial species. The growth of bacterial strains was inhibited at MIC = 0.31–0.63 mg/mL by most of these extracts. Bacteria belonging to Gram-positive cocci, i.e., *Staphylococcus epidermidis* ATCC 12228 and *Micrococcus luteus* ATCC 10240, were the most sensitive to the extracts from *M. didyma* (MIC = 0.07–0.15 mg/mL) and *M. citriodora* (MIC = 0.15 mg/mL). Susceptibility of *Staphylococcus aureus* strains and *Bacillus subtilis* ATCC 6633 to all extracts was comparable with MIC = 0.31–0.63 mg/mL. Other bacteria, *Bacillus cereus* ATCC 10876 and *Enterococcus faecalis* ATCC 29212, were slightly less sensitive to the extracts from *M. bradburiana, M. fistulosa* and *M. punctata* (MIC = 1.25 mg/mL). The majority of the extracts showed MBC comparable to MIC. Due to this, all extracts indicated bactericidal effect (MBC/MIC = 1–2) towards Gram-positive microorganisms. Moreover, luteolin (compound also present in the methanolic *Monarda* spp. extracts) was used as the reference antibacterial natural compound in this study. The obtained MIC values of luteolin for Gram-positive bacteria were 0.31–1.25 mg/mL.

Analyzing the results from Table 4, it was shown that the antimicrobial activity of the extracts from *Monarda* spp. against Gram-negative bacteria was lower (MIC = 2.5–10 mg/mL), except of *Bordetella bronchiseptica* ATCC 4617 (MIC = 0.63–1.25 mg/mL). All extracts also exhibited bactericidal effects towards these microorganisms (MBC/MIC = 1) since MIC and MBC values were the same for each bacterial species. MIC of luteolin was 10 mg/mL for all Gram-negative bacteria.

Moreover, the results presented in Table 5 indicated some antifungal effect of the tested extracts against yeasts belonging to *Candida* spp. (MIC = 1.25–10 mg/mL). *C. albicans* ATCC 10231 was found to be the most susceptible to extracts from *M. punctata*, *M. media, M. didyma* and *M. citriodora*, whereas *C. krusei* ATCC 14243 was most susceptible to the extract from *M. didyma* (MIC = 1.25 mg/mL). Similarly, as in the case of bacteria, all extracts showed fungicidal effect. MFC/MIC values ranged from 1 to 2 since MIC and MFC values were equal or almost equal for each yeast species. Luteolin was used also as the standard antifungal compound with MIC = 0.63–2.5 mg/mL for the yeast species.

## 4. Discussion

Many studies had been published concerning the elucidation of polyphenolic profiles of various Lamiaceae species through different chromatographic methods, usually HPLC [4,5,12,13]. However, there is scant information about polyphenols in *Monarda* spp. In this study, data on the profiles of polyphenols in the 70% (*v/v*) methanolic extracts of *M. citriodora*, *M. bradburiana*, *M. didyma*, *M. media*, *M. fistulosa* and *M. punctata* cultivated in Poland were obtained using HPLC–DAD–ESI/MS/MS. The composition of *Monarda* spp. proved to vary during plant phenology and in different environmental conditions. These discrepancies were observed by Gontar et al. [31], who described substantial changes in the terpene constituents contents in this gender. Based on these conclusions, the authors of this manuscript found it crucial to determine the polyphenolic fingerprint of the herein investigated plant samples. The selection of the extracting solvent was carried out according to the previously published protocols that proposed the usage of methanol:water mixtures in the recovery of phenolic compounds from plant matrix [32]. In this study, a total of 18 phenolic compounds were detected in these extracts including both phenolic acids and flavonoids. There were some differences between the composition of the extracts in the number and the type of phenolic compounds. All extracts contained 12 common compounds: protocatechuic acid, *p*-OH-benzoic acid, chlorogenic acid, coumarylquinic acid, rosmarinic acid, kaempherol-3-rutinoside, apigenin-7-rutinoside, luteolin-3-glucuronide, luteolin, apigenin, naringenin and naringin. The presence of six constituents (gallic acid, hydroxybenzoic acid glucoside, *p*-coumaric acid, ferulic acid, luteolin-7-glucoside and apigenin-7-glucoside) varied depending on the species of *Monarda*. The number of the identified compounds ranged from 14 to 17 in various species, with the extract of *M. fistulosa* being the one with the higher number of such compounds: 17 of them. As reported elsewhere, in the 70% (*v/v*) ethanolic extracts from fresh leaves and flowers of *M. fistulosa* using the LC-MS technique, only a few components were identified: apigenin-7-rutinoside, apigenin, linarin and rosmarinic acid [6]. Among these compounds, linarin was not detected in the methanolic extract of *M. fistulosa* in the present study.

There is also some literature data concerning studies on polyphenols in *Monarda* spp. by other chromatographic techniques. Davies and Mazza [13], using HPLC, showed the presence of anthocyanins, flavonoids and hydroxycinnamic acids, such as p-coumaric acid glucoside, flavone-7-O-glucoside, 7,4’-dihydroxyflavone 8-C-glucoside, 5-hydroxyflavone acylated pelargonidin glycosides, apigenin-7-O-glucoside and diacylated pelargonidin-3,5-diglucoside, in the extracts of *M. fistulosa* petals. Rosmarinic acid was found to be the main component of aqueous and methanolic extracts obtained from the *M. fistulosa* herb grown in Ukraine [4,5]. According to data presented elsewhere [11,12], the extracts from leaves and flowers of *M. didyma* grown in Lithuania were found to include polyacylated anthocyanins containing coumaric and malonic acids as well as the flavonols rutin, hyperoside, quercitrin, luteolin and quercetin revealed by HPLC. The authors noted that the amount of flavonoids was higher in the flowers than in the leaves. Moreover, rosmarinic acid and the flavonoids rutin, hyperoside, naringin and naringenin were previously identified in the *M. didyma* herb by other authors in the high-performance thin layer chromatography (HPTLC)-based assay [4]. It was also found that the total flavonoids contents calculated as the luteolin equivalent number in the *Monarda* species introduced in Russia were at the levels of 1.57% in *M. fistulosa*, 1.63% in *M. didyma* and 1.61% in *M. citriodora* [33].

The polyphenols identified in the *Monarda* species are important from a pharmacological point of view. They were proved to exhibit anti-inflammatory, antiradical, antinociceptive, and analgesic properties [5]. As the plant is perceived as an important source of biologically active constituents, future fingerprinting of different species of the gender as well as isolation of single constituents are of high importance to modern phytotherapy and phytochemistry.

It is known that plants are important sources of natural antioxidants, especially widely distributed polyphenols, including both phenolic acids and flavonoids [1,15,16,17,34,35,36,37]. As reported here, the total content of polyphenols (TPC) in the methanolic extracts from various species of *Monarda* was comparable. These extracts showed slightly different but high antioxidant potential measured by DPPH radical scavenging activity comparable to the Trolox solution. Among them, the extracts from *M. media* and *M. didyma* indicated better antiradical properties. Thompson et al. [6] studied the antioxidant capacity of 70% (*v/v*) ethanolic extracts from fresh leaves and fresh and senescent flowers of *M. fistulosa* by the DPPH method too. They found that the EC_50_ for these extracts was 0.062 mg/mL, 0.283 mg/mL and 0.250 mg/mL, respectively, indicating that the extract from fresh leaves of *M. fistulosa* demonstrated the strongest antioxidant activity. In turn, EC_50_ value = 0.164 mg/mL was shown in the present study for the methanolic extract of the dried flowering herbs of *M. fistulosa*. Other investigated *Monarda* species, i.e., *M. punctata, M. media, M. didyma, M. citriodora* and *M. bradburiana*, showed comparable antioxidant properties. The obtained results suggest that the studied *Monarda* species may serve as source of effective natural antioxidants.

In the present research, the antimicrobial activity of the *Monarda* spp. methanolic extracts was also estimated. Recently, the treatment of infectious diseases is often ineffective due to the increased resistance of microorganisms to antibiotics. This situation clearly highlights the need for the search for new antimicrobial agents. Natural products are among the most promising candidates because they have low toxicity, low environmental impact and a broad spectrum of action when compared to synthetic antimicrobial substances [38]. Therefore, both antibacterial and antifungal effects of the above-mentioned extracts from *Monarda* spp. were analyzed. The presented data showed the noteworthy activity of the extracts against the reference strains of Gram-positive bacteria (*Staphylococcus, Micrococcus, Enterococcus* and *Bacillus* species) with MIC ≤ 1.25 mg/mL. This is consistent with the literature data according to which the MIC ≤ 1 mg/mL for the plant extracts may indicate their good antibacterial activity, suggesting the need for their further research [39]. However, Kuete and Efferth [40] proposed other criteria to categorize the activity of the plant extracts against bacteria and fungi, i.e., significant (MIC < 100 μg/mL), moderate (100 < MIC ≤ 625 μg/mL) or weak (MIC > 625 μg/mL).

Generally, strains belonging to Gram-positive bacteria are usually more sensitive to the plant extracts than those belonging Gram-negative microorganisms possessing more complex structures of the cell outer surface, especially the lipopolysaccharides barrier, including the cell wall [41]. The same principle was also observed in this study: MIC for Gram-positive bacterial species were in the range of 0.07–1.25 mg/mL, whereas Gram-negative ones ranged from 0.63 to 10 mg/mL. All extracts exerted bactericidal effect (MBC/MIC = 1–2). The presented results also indicated some antifungal activity against the reference strains of *Candida* spp. with MIC = 1.25–10 mg/mL and fungicidal effect (MFC/MIC = 1–2). According to our data, the methanolic extracts from *Monarda* spp. showed interesting antimicrobial activity and the differences in activity potency between extracts were small, although it seems that the highest effect came from the extract obtained from *M. didyma*.

As far as the authors know, there are no previous reports about the antibacterial and antifungal activity of methanolic extracts from these plants, so it is not possible to further compare these results with other publications. However, in some research centers, numerous polyphenols, including the same that we found in our extracts from *Monarda* spp., were isolated and identified. Their antimicrobial potential was also assessed. Probably, they are responsible for the antibacterial and antifungal activity of the studied extracts [42]. There are some data about the biological effect of some phenolic components from *Monarda* spp. [6,7].

Among them, the flavonoids apigenin, naringin, naringenin, luteolin and luteolin-7-glucoside, as well as phenolic acids, such as chlorogenic, rosmarinic, ferulic and coumaric acids, should be listed. The level of sensitivity of the microbial species to these constituents strongly depends not only on the type of active compound but also on the selected strains [42,43,44,45,46,47,48,49,50]. Phenolic compounds show mainly antibacterial activity against Gram-positive microorganisms [44]; however, some of them displayed antimicrobial effect towards different pathogens (*S. aureus, B. subtilis, E. coli, K. pneumoniae, P. aeruginosa* and *S. typhimurium*) with an MIC value ranging from 10 to 100 μg/mL. It should be noted that the antimicrobial potential of the fungal endophytic extracts associated with *M. citriodora* was also assessed elsewhere [45].

According to some data [46], the selected polyphenols apigenin, naringin or chlorogenic acid were highly active towards both Gram-positive and Gram-negative bacteria. The MIC values for reference strains of *S. aureus* ATCC 25923, *E. faecalis* ATCC 29212, *E. coli* ATCC 35218 and *P. aeruginosa* ATCC 10145 reached 2–16 µg/mL, while the clinical strains ranged between 32 and 128 µg/mL or above this [35]. The results from reports in the literature [47] exhibited a great efficiency of apigenin against bacilli, e.g., *B. subtilis* (MIC = 4 µg/mL), staphylococci, e.g., *S. aureus* (MIC = 8 µg/mL) and the Gram-negative rods *E. coli* (MIC = 16 µg/mL) or *P. aeruginosa* (MIC = 64 µg/mL). The other investigations of this flavonoid showed the moderate and weak activity against *S. aureus* (MIC = 500–1000 µg/mL) or higher activity (MIC = 4–>128 µg/mL) [45]. In the case of methicillin-resistant *S. aureus* (MRSA) strains, the MIC values ranged from 3.9 to 15.6 mg/mL. It was also shown that the growth of the opportunistic yeast *C. albicans* was inhibited by apigenin at MIC = 5 mg/mL [48].

The next compound, naringenin, also identified in our study in all extracts, has intensive antibacterial activity especially against MRSA and streptococci. This effect was caused by a reduction in the fluidity of the hydrophilic and hydrophobic regions of inner and outer cellular membranes [42,44]. The results of Duda-Madej et al. [51] demonstrated different levels of susceptibility of some species of bacteria to naringenin, e.g., *Lactobacillus* spp. with an MIC value of 250 μg/mL, *S. aureus* with an MIC of 62.5 μg/mL, *S. typhimurium* and *E. coli* with MICs of 125 μg/mL. The other authors [52] showed a similar potential of activity for naringenin towards *E. coli, B. subtilis* and *S. aureus.* This compound was characterized as exhibiting moderate antibacterial activity against both tested Gram-positive strains with MIC of 200 µg/mL each but was ineffective against *E. coli* up to a concentration of 400 µg/mL. In turn, data of Mundlia et al. [53] demonstrated a stronger effect of this flavonoid at MIC values of 6.5, 24.5, 12.5, 12.5 and 23.5 µg/mL against reference *S. aureus*, *S. epidermidis*, *B. subtilis, E. coli* and *P. aeruginosa,* respectively. Moreover, naringenin at 100 and 200 μg/mL suppressed the second (bacterial adhesion) and third stages (biofilm maturation) of *S. mutans* biofilm formation. Therefore, according to some reports [51], these properties may be advantageous for dental applications, e.g., to prevent the *S. mutans*-based formation of biofilm that results in anti-caries properties when appropriately concentrated. However, the susceptibility of individual microorganisms to naringenin is slightly controversial. Previous studies indicated the lack of its antimicrobial activity [54].

Luteolin has been also assessed by other authors. Their results showed a slightly higher sensitivity of some clinical isolates of MRSA to luteolin (MIC = 31.2–62.5 µg/mL) than methicillin-sensitive *S. aureus* (MSSA) strains (MIC = 125 µg/mL) [34]. Data of Qian et al. [55] indicated satisfactory MIC and MBC values of luteolin against *S. aureus* (16–32 μg/mL and 32–64 μg/mL) and against *L. monocytogenes* (32–64 μg/mL and 64–128 μg/mL). This compound also had an inhibitory effect on the remaining Gram-positive bacteria (e.g., *Lactobacillus* spp., *Bacteroides lactis* and *E. faecalis*) but not on Gram-negative microorganisms [39]. The MICs of luteolin against *E. coli* strains were ≥ 200 μg/mL [56].

According to results presented by Puupponen-Pimiä et al. [39], sensitivity to the phenolic acids (coumaric, ferulic and chlorogenic) was found to differ significantly among the tested microorganisms. These compounds showed activity against Gram-negative bacteria (especially towards *E. coli*) at high concentrations (500 μg/well). Lactic acid bacteria (*Lactobacillus* spp. and *Bacteroides* spp.) were more resistant to these polyphenols. Their activity against reference *S. aureus* strains was also shown [43].

A recent review of the literature shows that chlorogenic acid (characteristic for all our extracts) has a broad spectrum of antimicrobial activity, but its effect is very diverse. The investigations of Adamczak et al. [46] reported the moderate or weak activity of this acid against *S. aureus* (MIC = 500–1000 µg/mL) and a strong effect towards *E. faecalis* (MIC = 64 µg/mL). For *S. aureus* and *E. coli*, its MIC values ranged from between 40 and 80 to 10,000 µg/mL. In the case of *E. coli*, moderate activity of chlorogenic acid (MIC = 500 µg/mL) was presented by some authors. The lack of significant influence of this substance on *P. aeruginosa* strains at the concentrations tested (MIC > 1000 µg/mL) [21] was also confirmed. Some studies proved that rosmarinic acid (like chlorogenic acid found in our extracts) has an antimicrobial effect on Gram-positive and Gram-negative bacteria but the level of this activity was as follows: on *E. coli* (MIC > 250 µg/mL), *Bacillus* spp., *S. epidermidis* and *S. pyogenes* (MIC > 500 µg/mL). Other results demonstrated that the MIC values of this acid against *S. aureus* and MRSA were 0.8 and 10 mg/mL, respectively [46].

Several studies [57,58] also reported antimicrobial activity of gallic acid against several pathogens, such as *S. typhimurium, E. coli, S. aureus, Listeria innocua, Helicobacter pylori, Campylobacter* spp., *Pseudomonas* spp. or *Candida* spp. The studies performed by Sorrentino et al. [57] showed its antibacterial effect towards different species of *Pseudomonas* (*P. putida, P. fluorescens* and *P. fragi*) with MIC at 2.5–5 mg/mL and MBC at 10 mg/mL. Gallic acid was also suggested as a potential antimicrobial agent towards *Campylobacter* spp. The MIC values of gallic acid against *Campylobacter jejuni* and *Campylobacter coli* strains ranged from 15.63 to 250 μg/mL [58]. Moreover, gallic acid was active towards *Candida* strains, with MICs between 12.5 and 100 μg/mL. The most sensitive *Candida* species was *C. albicans* (MIC = 12.5 μg/mL), and the most susceptible filamentous fungus—*Trichophyton rubrum* (MIC = 43.75 μg/mL) [59]. In the case of protocatechuic acid, its antibacterial effect was examined against food spoilage bacteria, e.g., *S. typhimurium, E. coli, L. monocytogenes, S. aureus* and *B. cereus*. Its MIC values towards these bacteria were in the range of 24–44 μg/mL [60]. In turn, the ferulic acid was less active against *Enterobacter sakazakii* strains with MIC ranging from 2.5 to 5.0 mg/mL [61].

Moreover, according to the studies of Bouarab-Chibane et al. [43], the six foodborne pathogenic or food-spoiling bacterial species can be ranked by decreasing susceptibility to the 35 different polyphenols: *Listeria monocytogenes* (57.1%) > *B. subtilis, Salmonella enteritidis* and *S. aureus* (45.7% for these three bacterial strains) > *E. coli* (31.4%) > *P. aeruginosa* (17.1%).

The mechanisms of action of polyphenols are not yet fully understood but are known to involve many sites of action at the cellular level [43], including inhibition of cytoplasmic membrane function, influence on the biofilm formation, permeability, interaction with some crucial enzymes, inhibition of the DNA gyrase, synthesis of nucleic acids (DNA and RNA) and proteins, formation of a complex with proteins through nonspecific forces such as hydrogen bonding and hydrophobic effects, ability to inactivate adhesins, enzymes, cell envelope transport proteins and suppression of virulence factors such as toxins [44,45,46,47]. In the case of Gram-positive bacteria, intracellular pH modification as well as interference with the energy (ATP) generating system were also reported [43]. Moreover, the antimicrobial activities of many of them use different modes of action than those of conventional drugs, and thus could be of importance in the enhancement of antimicrobial therapy. Some polyphenols also manifest an ability to reverse the antibiotic resistance and enhance the action of the current antibiotics [42]. What is more, besides direct antibacterial activity, some polyphenols can exert a synergistic effect when combined with common antibiotics [45].

According to some literature data [62], EOs from different *Monarda* species also showed a wide range of antimicrobial activity towards both Gram-positive and Gram-negative bacteria and fungi. Their major components were primarily responsible for this activity: thymol, carvacrol, α-terpinene, ƴ-terpinene, p-cymene, geraniol, geranial, 1,8-cineole, α-phellandrene, β-caryophyllene, citral, limonene or cis-verbenol [18]. We also plan to research *Monarda* spp. EOs in the future.

The selected data on the antimicrobial effect of only some polyphenols may confirm the relationship between their presence in methanolic extracts from *Monarda* spp. and their bioactivity towards microorganisms, especially Gram-positive bacteria.

## 5. Conclusions

The present study analyzed the phenolic compounds in 70% (*v/v*) methanolic extract from flowering herbs of *M. bradburiana*, *M. citriodora*, *M. didyma*, *M. fistulosa*, *M. media* and *M. punctata*. Eighteen compounds were identified by RP-HPLC/ESI-QTOF/MS analysis. The profiles of polyphenols included the common compounds and those dependent on the plant species. The phenolic compounds, i.e., chlorogenic and ferulic acid that were presented in the investigated species, greatly contribute to the antioxidant activity of extracts of *Monarda* spp., which can be an alternative antioxidant for use in food. The ability to scavenge free radicals by a methanol–water extract from these plants was determined for the first time. The obtained results showed that the investigated extracts from various species of *Monarda* possessed high antioxidant potential. These extracts also indicated noteworthy activity against Gram-positive bacteria, both opportunistic and pathogenic species, constituting microbiota of the human body. Based on these novel data, *Monarda* spp. seems to be an important reservoir of natural antioxidant and antibacterial agents, especially active against staphylococci.

## Figures and Tables

**Table 1:** Analysis of phenolic compounds by HPLC–DAD–ESI-QTOF-/MS/MS in 70% (*v/v*) methanolic extracts of *Monarda* species. (Rt—retention time, *—the components that were identified also based on a direct comparison with standards).

No.	Rt [min]	Phenolic Compounds	Empirical Formula	[M − H]^−^ (*m*/*z*)	Fragment Ions (*m*/*z)*	Diff (ppm)	*M. b.*	*M. c.*	*M. d.*	*M. f.*	*M. m.*	*M. p.*
1	4.25	gallic acid	C_7_H_5_O_5_^−^	169.0100	125.0185	−2.08	+	+	+	+	+	−
2	9.16	protocatechuic acid *	C_7_H_5_O_4_^−^	153.0217	109.0279	−9.53	+	+	+	+	+	+
3	12.72	hydroxybenzoic acid glucoside	C_13_H_15_O_8_^−^	299.0784	137.0252	−3.86	+	+	+	+	+	−
4	15.63	*p*-OH-benzoic acid	C_7_H_5_O_3_^−^	137.0259	108.0218	−8.57	+	+	+	+	+	+
5	18.78	chlorogenic acid	C_16_H_17_O_9_^−^	353.0859	191.0532; 179.0339; 173.0405; 135.0454	−7.61	+	+	+	+	+	+
6	20.47	coumaryl-quinic acid *	C_16_H_17_O_8_^−^	337.0962	191.0590; 173.0484; 163.0414	−8.01	+	+	+	+	+	+
7	23.91	*p*-coumaric acid	C_9_H_7_O_3_^−^	163.0369	119.0480	−8.12	+	+	+	+	−	−
8	27.40	kaempherol-3-rutinoside	C_27_H_29_O_15_^−^	593.1445	285.0403	−7.25	−	+	−	+	−	+
9	28.84	luteolin-7-glucoside	C_21_H_19_O_11_^−^	447.0965	285.0555	−5.39	+	+	+	+	+	+
10	28.96	naringin	C_27_H_31_O_14_^−^	579.1739	271.0586	−7.88	+	+	+	+	+	+
11	29.41	ferulic acid *	C_10_H_9_O_4_^−^	193.0474	178.0205; 149.0590	−11.69	+	−	−	−	−	+
12	29.49	apigenin-7-rutinoside	C_27_H_29_O_14_^−^	577.1597	269.0492	−5.92	+	+	+	+	+	+
13	30.01	apigenin-7-glucoside	C_21_ H_19_ O_10_^−^	431.0970	269.0490	3.17	−	−	+	+	−	+
14	30.12	luteolin-3-glucuronide *	C_21_H_17_O_12_^−^	461.0725	285.0404	0.11	+	+	+	+	+	+
15	31.74	rosmarinic acid	C_18_H_15_ O_8_^−^	359.0756	197.0431; 161,0228	−9.05	+	+	+	+	+	+
16	35.37	luteolin *	C_15_H_9_O_6_^−^	285.0430	267.7263	−8.87	+	+	+	+	+	+
17	37.23	naringenin	C_15_H_11_O_5_^−^	271.0603	151.0088; 119.0276; 107.0412	3.30	+	+	+	+	+	+
18	37.47	apigenin	C_15_H_9_O_5_^−^	269.0469	117.0338	−0.94	+	+	+	+	+	+

*M. b.*—*Monarda bradburiana* L.C. Beck; *M. c.*—*Monarda citriodora* Cerv. ex Lag.; *M. d.*—*Monarda didyma* L.; *M. f.*—*Monarda fistulosa* L.; *M. m.*—*Monarda media* Willd.; *M. p.*—*Monarda punctata* L.

**Table 2:** Antimicrobial activity of 70% (*v/v*) methanolic extracts of *Monarda* species by diffusion method based on growth inhibition zone diameters (mm).

Species	Zone of Growth Inhibition (mm)					
	*M. bradburiana*	*M. citriodora*	*M. didyma*	*M. fistulosa*	*M. media*	*M. punctata*
Gram-positive bacteria						
*Staphylococcus aureus* ATCC 25923	+/−	13	16	+/−	11	–
*Staphylococcus aureus* ATCC 6538	10	12	15	–	8	8
*Staphylococcus aureus* ATCC 29213	–	12	14	–	11	–
*Staphylococcus aureus* ATCC 43300	9	14	15	–	11	11
*Staphylococcus epidermidis* ATCC 12228	–	–	13	–	11	10
*Micrococcus luteus* ATCC 10240	11	15	15	–	12	10
*Bacillus subtilis* ATCC 6633	20	31	35	16	30	29
*Bacillus cereus* ATCC 10876	14	17	17	13	14	14
*Enterococcus faecalis* ATCC 29212	–	11	11	–	12	+/−
Gram-negative bacteria						
*Bordetella bronchiseptica* ATCC 4617	13	18	19	13	19	16
*Klebsiella pneumoniae* ATCC 13883	+/−	–	13	–	12	+/−
*Proteus mirabilis* ATCC 12453	–	–	11	–	10	–
*Salmonella* Typhimurium ATCC 14028	10	11	13	–	12	11
*Escherichia coli* ATCC 25922	–	–	+/−	–	10	+/−
*Pseudomonas aeruginosa* ATCC 27853	13	13	13	13	14	12
Fungi						
*Candida albicans* ATCC 2091	13	12	12	13	12	13
*Candida albicans* ATCC 10231	+/−	+/−	16	+/−	16	14
*Candida parapsilosis* ATCC 22019	+/−	+/−	+/−	+/−	+/−	+/−
*Candida glabrata* ATCC 90030	–	–	–	–	–	–
*Candida krusei* ATCC 14243	11	12	15	10	14	15

– no zone of growth inhibition; +/− growth of single colonies in the growth inhibition zone or slight growth nears around the wells.

**Table 3:** Antimicrobial activity in vitro of 70% (*v/v*) methanolic extracts of *Monarda* species expressed as MIC and MBC (mg/mL) values against the reference strains of Gram-positive bacteria. The representative values (mode) are presented.

Species	*M. bradburiana*		*M. citriodora*		*M. didyma*		*M. fistulosa*		*M. media*		*M. punctata*	
	MIC	MBC	MIC	MBC	MIC	MBC	MIC	MBC	MIC	MBC	MIC	MBC
*S. aureus* ATCC 25923	0.63	0.63	0.31	0.31	0.31	0.31	0.63	0.63	0.31	0.31	0.31	0.31
*S. aureus* ATCC 6538	0.63	0.63	0.31	0.31	0.31	0.31	0.31	0.31	0.31	0.31	0.63	0.63
*S. aureus* ATCC 29213	0.63	0.63	0.31	0.31	0.31	0.31	0.63	0.63	0.31	0.31	0.31	0.31
*S. aureus* ATCC 43300	0.63	0.63	0.31	0.31	0.31	0.31	0.31	0.63	0.31	0.31	0.31	0.31
*S. epidermidis* ATCC 12228	0.31	0.31	0.15	0.15	0.15	0.15	0.31	0.63	0.15	0.31	0.31	0.31
*M. luteus* ATCC 10240	0.63	0.63	0.31	0.31	0.07	0.15	0.63	0.63	0.31	0.31	0.31	0.31
*B. subtilis* ATCC 6633	0.63	0.63	0.31	0.31	0.31	0.31	0.63	0.63	0.63	0.63	0.63	0.63
*B. cereus* ATCC 10876	0.63	0.63	0.63	0.63	0.31	0.31	1.25	1.25	0.63	0.63	1.25	1.25
*E. faecalis* ATCC 29212	1.25	1.25	0.63	0.63	0.31	0.63	1.25	1.25	0.63	0.63	0.63	0.63

**Table 4:** Antimicrobial activity in vitro of 70% (*v/v*) methanolic extracts of *Monarda* species expressed as MIC and MBC (mg/mL) values against the reference strains of Gram-negative bacteria. The representative values (mode) are presented.

Species	*M. bradburiana*		*M. citriodora*		*M. didyma*		*M. fistulosa*		*M. media*		*M. punctata*	
	MIC	MBC	MIC	MBC	MIC	MBC	MIC	MBC	MIC	MBC	MIC	MBC
*B. bronchiseptica* ATCC 4617	1.25	1.25	0.63	0.63	2.5	2.5	1.25	1.25	2.5	2.5	1.25	1.25
*K. pneumoniae* ATCC 13883	5	5	10	10	2.5	2.5	10	10	2.5	2.5	10	10
*P. mirabilis* ATCC 12453	10	10	10	10	5	5	10	10	5	5	10	10
*S. Typhimurium* ATCC 14028	10	10	10	10	5	5	10	10	5	5	10	10
*E. coli* ATCC 25922	10	10	10	10	5	5	10	10	10	10	10	10
*P. aeruginosa* ATCC 27853	10	10	5	5	5	5	10	10	5	5	10	10

**Table 5:** Antimicrobial activity in vitro of 70% (*v/v*) methanolic extracts of *Monarda* species expressed as MIC and MFC [mg/mL] values against the reference strains of fungi. The representative values (mode) are presented.

Species	*M. bradburiana*		*M. citriodora*		*M. didyma*		*M. fistulosa*		*M. media*		*M. punctata*	
	MIC	MBC	MIC	MBC	MIC	MBC	MIC	MBC	MIC	MBC	MIC	MBC
*C. albicans* ATCC 2091	2.5	5	2.5	2.5	2.5	2.5	2.5	5	2.5	5	5	5
*C. albicans* ATCC 10231	2.5	5	1.25	2.5	1.25	2.5	2.5	5	1.25	2.5	1.25	2.5
*C. parapsilosis* ATCC 22019	5	5	2.5	5	2.5	5	5	5	2.5	5	2.5	5
*C. glabrata* ATCC 90030	5	10	2.5	2.5	2.5	5	5	10	5	5	5	5
*C. krusei* ATCC 14243	5	5	2.5	2.5	1.25	2.5	5	5	2.5	5	2.5	5

## Data Availability

Not applicable.

## Supplementary Materials

The following supporting information can be downloaded at: https://www.mdpi.com/article/10.3390/pharmaceutics15030964/s1. Table S1. Fragmentation data of the tentatively identified components for polyphenolic compounds identified in 70% (*v/v*) MeOH in extracts of tested species of *Monarda* L. Table S2. Mass chromatogram of the total extract TIC together with ion chromatograms of all identified phenolic acids in the tested species of *Monarda* L. Figure S1. Antimicrobial activity of 70% (*v/v*) methanolic extracts of *Monarda* species assessed by diffusion method based on growth inhibition zone diameters for the selected bacterial species.
