# Supplementary material for: Comparative HPLC–DAD–ESI-QTOF/MS/MS Analysis of Bioactive Phenolic Compounds Content in the Methanolic Extracts from Flowering Herbs of Monarda Species and Their Free Radical Scavenging and Antimicrobial Activities
Source: Pharmaceutics. 2023 Mar 16;15(3):964. doi: 10.3390/pharmaceutics15030964 (PMC10053500; doi:10.3390/pharmaceutics15030964)

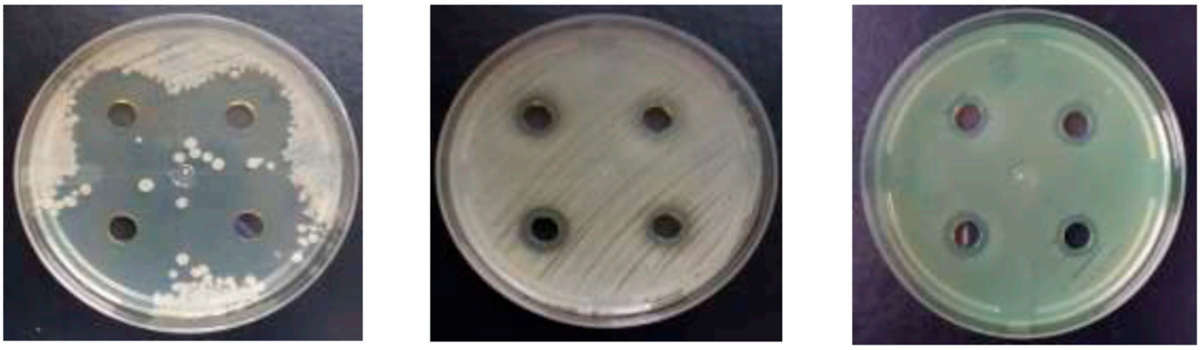

a) *Bacillus subtilis* ATCC 6633

b) *Staphylococcus aureus* ATCC 29213

c) *Pseudomonas aeruginosa* ATCC 27853

Figure S1. Antimicrobial activity of 70% (v/v) methanolic extracts of *Monarda* species assessed by diffusion method based on growth inhibition zone diameters for the selected bacterial species.

Table S1. Fragmentation data of the tentatively identified components for polyphenolic compounds identified in 70% (v / v) MeOH in extracts of tested species of *Monarda* L.

gallic acid

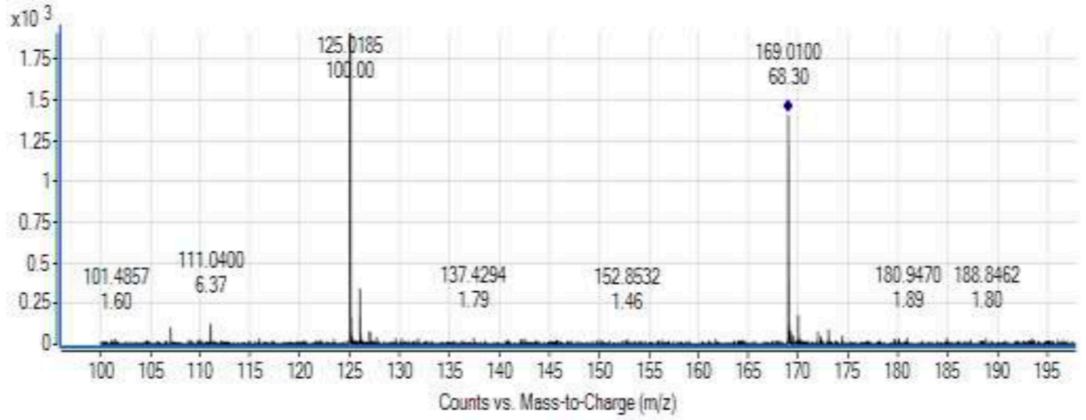

protocatechuic acid

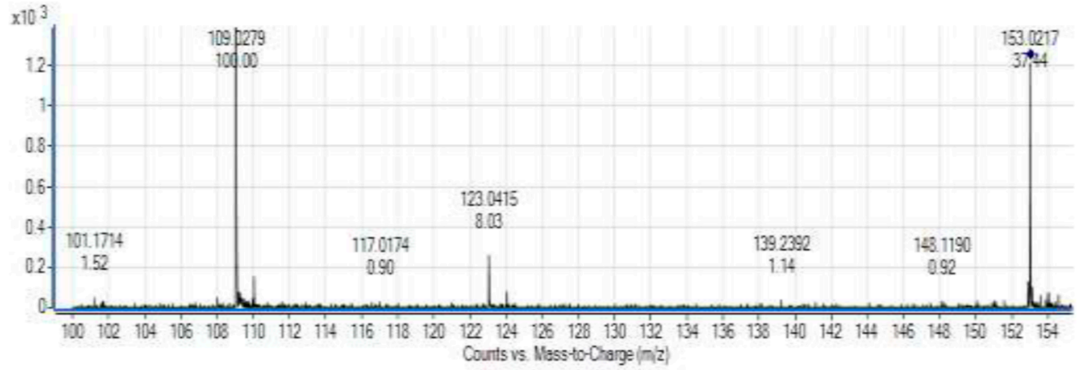

hydroxybenzoic acid glucoside

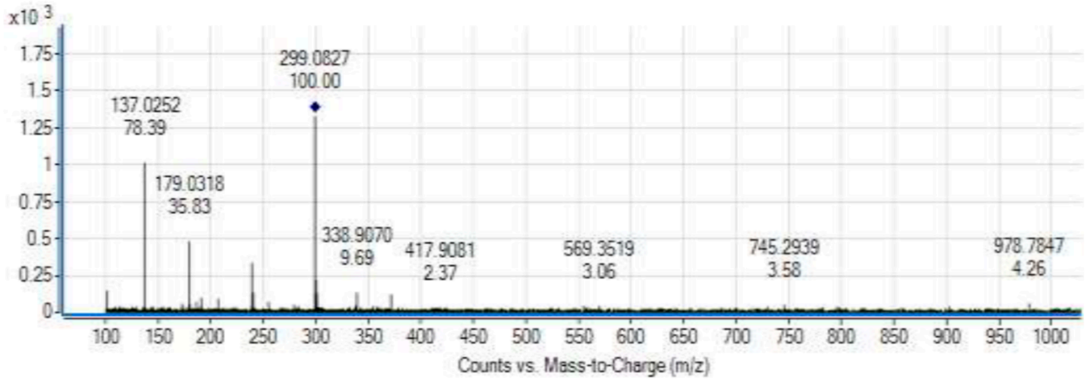

***p*-OH-benzoic acid**

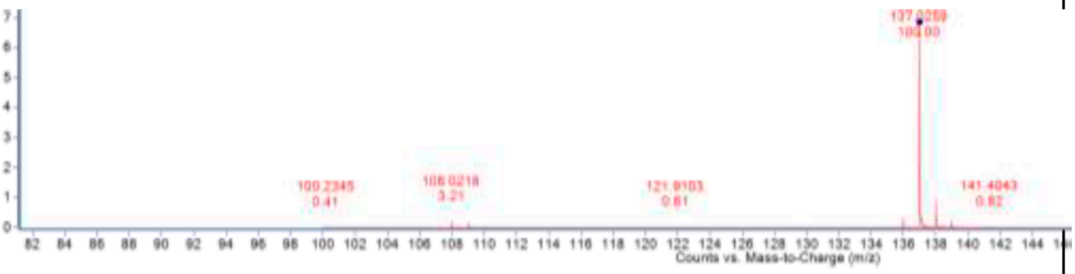

chlorogenic acid

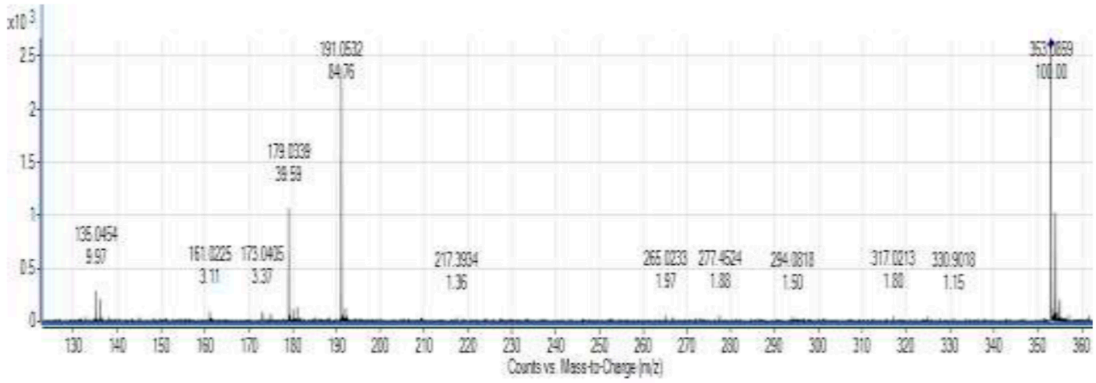

coumarylquinic acid

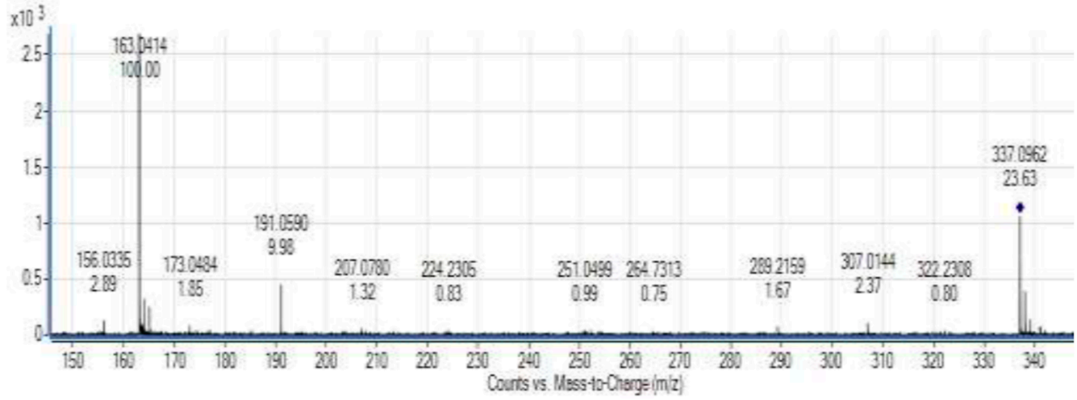

***p*-coumaric acid**

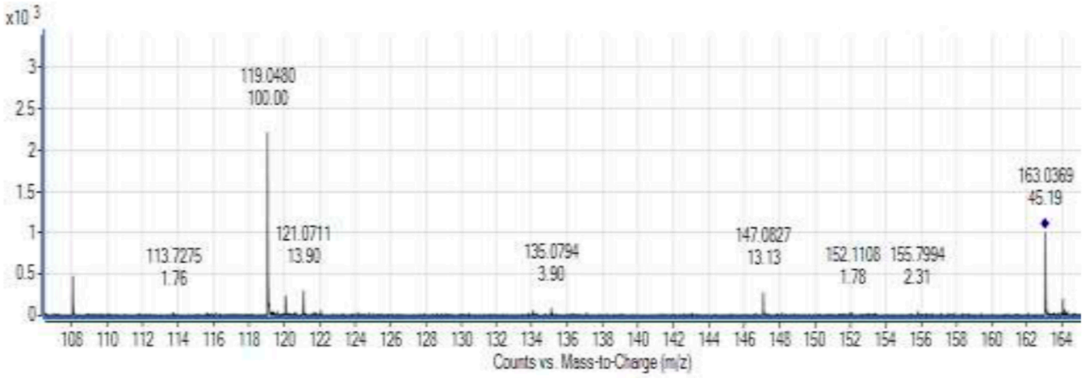

luteolin-7-glucoside	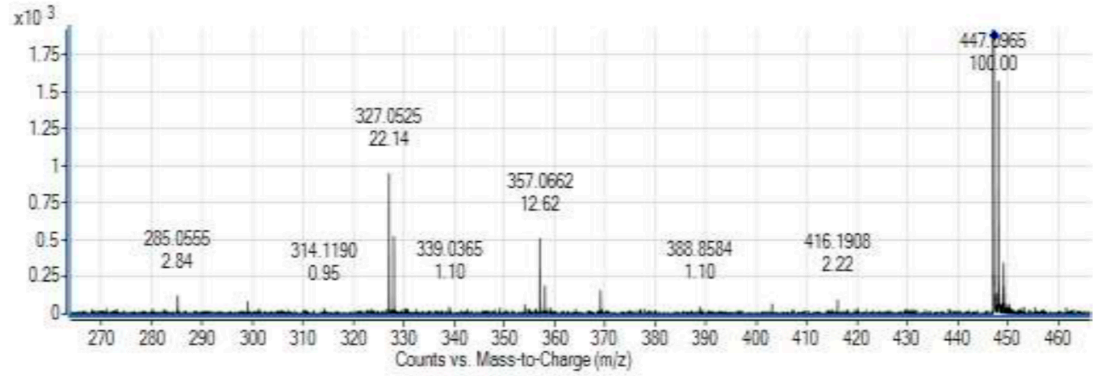
kaempherol-3-rutinoside	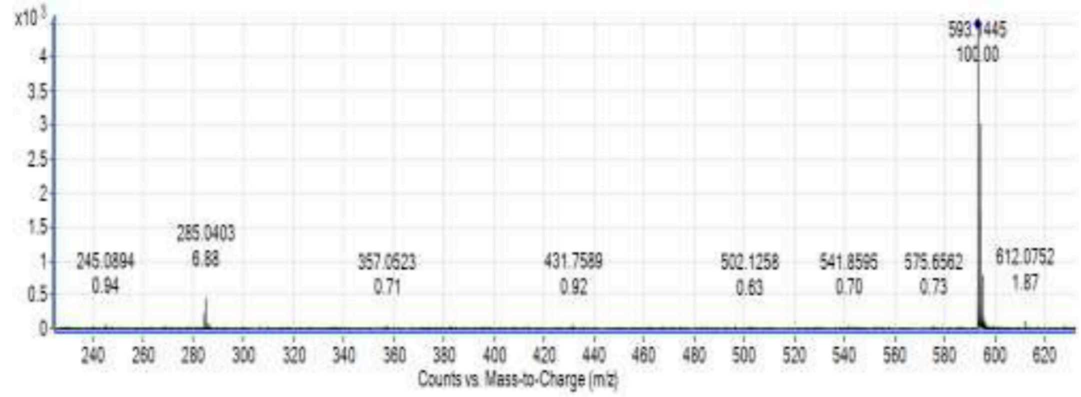
naringin	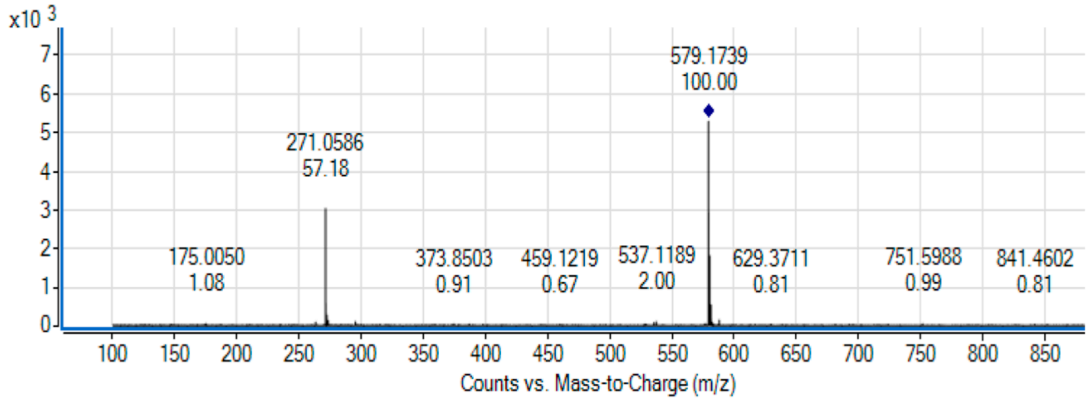
ferulic acid	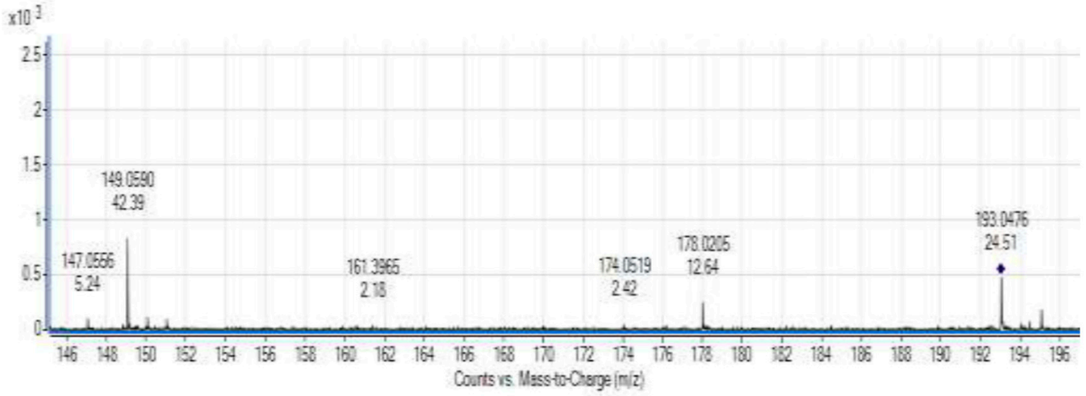

Table S2. Mass chromatogram of the total extract TIC together with ion chromatograms of all identified phenolic acids in the tested species of *Monarda* L.

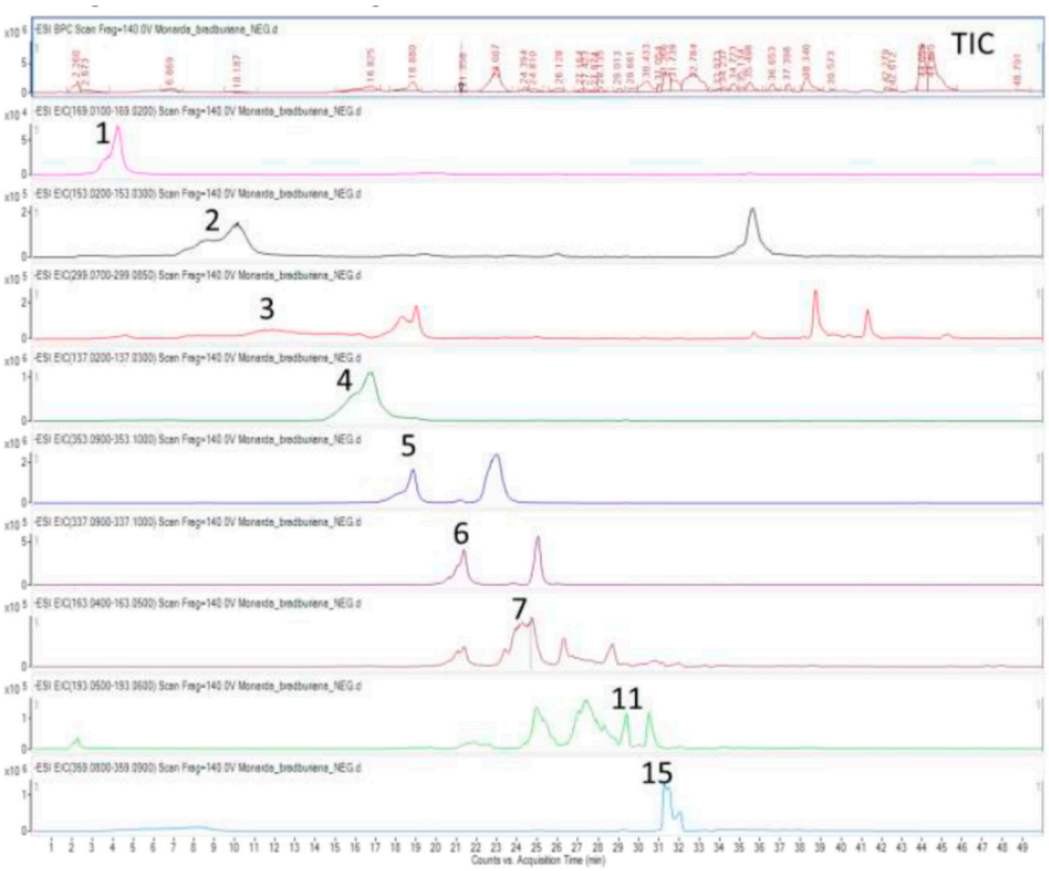

Supplement: Supplementary file 1 [file pharmaceutics-15-00964-s001.zip › pharmaceutics-2202874-supplementary.pdf]
